# Cognitive sovereignty and neuro-justice: neurotechnological advancements and mental autonomy in the Global South

**DOI:** 10.3389/fpubh.2026.1877249

**Published:** 2026-06-02

**Authors:** Chiara Saracini

**Affiliations:** The Neuropsychology and Cognitive Neurosciences Research Center, Faculty of Health Sciences, Catholic University of Maule, Talca, Chile

**Keywords:** artificial intelligence, cognitive sovereignty, global south, indirect neurotechnology, neuroenhancement, neuro-justice, neurorights, neurotechnology

## 1 Introduction

In recent years, the development of neurotechnologies has stimulated extensive ethical and legal debates around the concepts of neurorights, cognitive liberty, and mental privacy (1). Yet, the current debate has been shaped largely by a technocratic logic originating in Global North contexts, where neurotechnological innovation is often framed in terms of cognitive enhancement and human optimization (2, 3). This perspective overlooks the material and ontological realities of the Global South, where the priority is not the “upgrade” of a functioning system, but the fundamental right to basic healthcare and neurorehabilitation.

In a moment when Brain Capital (the cognitive, emotional and mental health resources of populations, (4, 5)) is increasingly recognized as a critical determinant of national development and resilience, its equitable distribution becomes a public health imperative. Yet the current technological race toward enhancement risks producing the opposite outcome for the South: those left behind will not only never claim any neuroright, but will likely see their actual brain capital drained rather than “improved”.

Furthermore, the integration of Artificial Intelligence (AI) into neurotechnology adds a layer of complexity (6, 7). The current trend is to shift from simple interfaces able to “read” brain signals toward agentic and multimodal systems that actively and adaptively interact with the user's brain. This shift from a physical device to an adaptive, AI-powered ecosystem that collects, processes, and returns data deserves a reframing of the very concept of neurotechnology. The integration of AI into our everyday technologies has blurred the lines between direct brain stimulation and indirect cognitive modulation, possibly altering states of consciousness and influencing affective cognition and even agency through simulated empathic, responsive and data-driven personalization (8). The current notion of “neurotechnology” thus appears incomplete, unable to capture the relational consequences of indirect (not only physical) stimulation of the neural substrate (7, 9).

The very framing of the debate differs across geographies. In the Global North, the discussion centers on the freedom to enhance and on regulating neurotechnologies to protect users' autonomy and privacy. In the Global South, the central problem is more elementary: building public health systems robust enough to grant patients access to care, whether basic or technologically mediated. Although Southern countries have actively participated in the neurorights debate [Chile being the most prominent example, (39)] they remain structurally distant from the economic, technological and scientific centers that still dominate the global scene. Rather than developing their own innovations competitively, they tend to find themselves on the receiving end of digital and data colonialism (10, 11), or supplying low-cost labor for the big tech companies of countries with consolidated technological traditions.

In this opinion paper, I argue that a broad concept of neuro-justice can function as a bioethical framework that extends beyond the legalistic “neurorights” approach to ensure distributive equity and the protection of cognitive sovereignty, integrating currently fragmented strands of debate: distributive-justice critiques of cognitive enhancement (12–14) and decolonial perspectives from the Global South (15–17). This principle should guide each country's scientific and technological efforts to prioritize neurotechnologies aimed at addressing clinical needs first, helping to reduce structural asymmetries in global scientific and technological development. The shift required is from a competitive logic to a collaborative one, first ensuring an adequate level of functioning for all, then developing technologies that build on what is already known to work.

## 2 From enhancement to distributive neuro-justice

Cognitive or neuroenhancement (CNE) refers to the use of technology to augment cognitive functions in healthy populations (18). The ethical debate around CNE has been primarily constructed around technocratic ideals: some positions support the improvement of “normal” cognitive functions to achieve superior performance and pursue national wealth in already-rich countries, a concept known as cognitive capitalism (19), while others frame the tension between the freedom to enhance and the equality of those who cannot afford it as a problem of distributive justice (12, 20).

From a Global South perspective, however, this focus appears not only premature but ethically skewed. In Africa, Latin America, or parts of Asia, the most pressing challenge is not the enhancement of an already-healthy elite, but the lack of equitable access to basic neurorehabilitation and neurological healthcare. Limited educational and health resources may further exacerbate neurological vulnerabilities tied to poverty, trauma, and inequality (21, 22), creating a widening gap that risks becoming insurmountable, a “neuro-stratified” society where biological and cognitive inequalities are permanently crystallized by technology (23).

This stratification is not only political but epistemic. Most neurotechnological tools and their underlying algorithms are calibrated on data from WEIRD populations [Western, Educated, Industrialized, Rich, and Democratic, (24)], which represent only a small fraction of humanity, while early adversity and socioeconomic deprivation are known to induce structural brain differences in areas related to executive function and emotional regulation (22, 25). Applying enhancement “templates” calibrated on brains that have never experienced such deprivation to marginalized populations is therefore not just scientifically questionable, but bioethically irresponsible. The same logic extends to cultural diversity: ignoring the cosmovisions of indigenous peoples [e.g., Ubuntu from Africa, (15, 26)] regarding relationality and the collective mind is a loss for science and reinforces a dominant paradigm that has fueled global biocultural homogenization (27), undermining the epistemic justice and cultural sensitivity that recent scholarship has called for in the Global South (28, 29).

These concerns extend earlier critiques from Global South neuroethics, which have argued that mainstream debates on neurorights and CNE have neglected practical justice for diverse communities and ignored the colonial legacy of race-and-intelligence research that still shapes neurotechnological development (16, 17, 30, 31). Building on these contributions and on the Rawlsian framing of cognitive enhancement and inequality (12–14), I suggest that a framework of Neuro-Justice must prioritize distributive equity, shifting the focus from “boosting” the few to “restoring” the many; improvement can still occur later. While “neuro-justice” has traditionally been used within neurolaw (32), I propose a shift toward a bioethical sense, focused on the equitable protection of cognitive sovereignty and mental autonomy in the Global South to avoid new forms of coloniality [a sort of “neuro-capitalism”, (33)].

## 3 Indirect neurotechnology and the erosion of cognitive sovereignty

The “neurorights” movement (1, 18) is a significant milestone in legal philosophy, but its framework has been outpaced by the rapid pace of technological development. By focusing almost exclusively on direct brain–machine interfaces and the protection of “neural data”, these legal instruments fail to address the broader spectrum of technological influences that already jeopardize cognitive sovereignty and mental autonomy without ever physically touching the brain.

The very definition of “neurotechnology” is therefore incomplete. In the digital age, information itself, when delivered through adaptive, scalable, and highly personalized algorithms, acts as an “indirect neurotechnology”: AI-driven platforms, social media, and immersive digital environments modulate attention, emotional states, and decision-making in ways that mirror some effects of direct brain stimulation, suggesting that neuroethical frameworks focused on direct interventions are insufficient to address the broader informational conditions under which mental autonomy is exercised (7–9).

Unlike traditional cultural influences such as literature or cinema, these systems are dynamically adaptive and reactive, creating continuous feedback loops that erode the boundary between autonomous choice and external conditioning. AI does not need a chip to “enter” the mind: if CNE is defined as the alteration of cognitive states to improve performance, then generative AI with personalized algorithms (acting as “lenses” that modify the perception of reality) and AI-driven informational environments may produce forms of indirect cognitive modulation that challenge current neuroethical frameworks. The risk is a progressive erosion of cognitive sovereignty: not just a problem of privacy, but of agency (7). Predictive algorithms decide before us, transforming CNE into a conditioning process rather than an enhancement.

This raises a question that goes beyond legal compliance: can the right to enhance justify systems that progressively constrain the conditions of free agency? Without accounting for the indirect ways in which autonomy is compromised (even before AI-adaptive neurochips reach widespread use) we leave a substantial ethical loophole. Cognitive sovereignty must be understood not just as the right to a “private brain”, but as the right to control one's own cognitive processes and resist manipulation from any technological environment that modifies perspective and agency without conscious consent.

## 4 Cognitive extractivism and data colonialism

The “problem” of neurotechnology and neurorights, as currently framed, is largely a problem for those who already have enough resources to debate user rights. In the South, the problem hardly arises in the same form: the actors who can afford to develop neurotechnologies for enhancement are concentrated in the North, while the South often lacks even the basic technologies to address public health needs. The question is unavoidable: who benefits from neurotechnological progress?

Drawing on Couldry and Mejias (10), Big Tech multinationals extract biometric and cognitive data from the South (where regulations are weak) to train AI models then deployed in the North. This is a critical geo-economic problem: a perpetuation of imperialist exploitative regimes, now in the form of “data extractivism” (11) or, more specifically, cognitive extractivism (34). Recent policy analyses similarly identify three reinforcing dimensions of digital asymmetry: infrastructural dependency, epistemic injustice, and economic extraction, and call for “digital sovereignty” as a structural counterpart to data colonialism (35). Extending this logic to the neural domain, the analogous goal is cognitive sovereignty, encompassing not only the control of brain data but also the development of locally anchored neurotechnological infrastructures.

The labor required to make these systems functional is also extracted from the South: data labelers in Kenya, Venezuela, and the Philippines are paid 1–2 USD per hour to annotate sensitive content under exploitative conditions, creating a “global underclass of digital workers” (36, 37).

Without a framework of Neuro-Justice that prioritizes distributive equity and algorithmic justice and responsibility, neurotechnology risks becoming a tool for cognitive extractivism and data colonialism rather than human flourishing (10, 11, 38).

## 5 Discussion

The rapid advancement of neurotechnology, amplified by the predictive power of AI, has outpaced existing legal and ethical frameworks. The current “neurorights” discourse, while a necessary first step, is insufficient to address the unique vulnerabilities of the Global South. Reform must move beyond the protection of neural data toward a broader commitment to neuro-justice and cognitive sovereignty: a shift from a reactive ethics, responding to technology only once deployed, to a proactive framework that permeates technological development from inception. Legally, this means establishing a right to cognitive sovereignty that guarantees control over one's own informational and mental environments, resisting both direct neural intervention and the indirect cognitive modulation and extractivism that characterize the digital age.

Scientific and social equity are equally at stake. If neurotechnologies remain calibrated on a narrow, privileged subset of humanity, they risk becoming instruments of further exclusion: the Global South must not be treated as a mere source of “biased” data or as a testing ground, and the benefits of neuroscientific progress should be distributed according to the principle of justice rather than market logic.

Neurorights and neurotechnologies, as currently conceived, are largely embedded in a neoliberal, “neuro-capitalist” framework—made by WEIRD people for WEIRD people who already hold an advantage. Without a clear ethical purpose of neuro-justice, further development will only widen the imbalance and reinforce neuro-inequality. In a world facing a multi-crisis of economic, climate, and social instability, if the answer to the question “who benefits from advances in neurotechnologies?” remains limited to a global elite while the majority faces an erosion of mental autonomy and an extraction of their cognitive essence, then the promise of neurotechnology risks reinforcing, rather than dismantling, existing structures of inequality.
